# Changes in vital signs, ventilation mode, and catecholamine use during intensive care aeromedical evacuation flights

**DOI:** 10.3389/fpubh.2023.1100832

**Published:** 2023-02-27

**Authors:** Janina Post, Marc Christoph Maeckelburg, Vitali Jagel, Stefan Sammito

**Affiliations:** ^1^Department Experimental Aerospace Medicine Research, German Air Force Centre of Aerospace Medicine, Cologne, Germany; ^2^Department of Occupational Medicine, Faculty of Medicine, Otto von Guericke University of Magdeburg, Magdeburg, Germany

**Keywords:** aeromedical evacuation (AE), intensive care, disaster medicine, military medicine, ventilation

## Abstract

**Introduction:**

Especially after (natural) disasters, local health systems are also destroyed or their ability to work is massively restricted. The transport of injured patients is therefore often necessary for further care. Numerous nations keep fixed-wing aircraft with intensive-care capabilities available for secondary transport, but little data on the transport is available to date.

**Methods:**

An analysis of all flights with the German Air Force's intensive care fixed-wing-aircraft carried out in the context of humanitarian aid missions since 2002 with a focus on intubated patients was done.

**Results:**

A total of 38 patients were transported. Two patients had to be intubated on or during transport. There were significant changes in the necessary positive end-expiratory pressure (PEEP) and the fraction of inspired oxygen. Circulatory parameters did not change.

**Discussion:**

Overall, there are no clinically relevant deteriorations due to secondary transport with corresponding air transfers. Due to the hypobaric hypoxic conditions on board of all aircrafts, intubation in clinically borderline patients should be performed electively on the ground before flight.

## 1. Introduction

The transport of patients requiring intensive care represents a major organizational challenge for medical crews which is also fraught with risk (1–3). Often, complications and a deterioration of the patient's circulatory condition occur during secondary transports (4–6). Numerous findings and recommendations are available for intensive care patients transported on the ground, by helicopter or fixed-wing aircraft (7–9), which included the compilation respectively the medical crew and the equipment on board, patient examination and preparation before transport/flight, task during transport/flight and contraindications for a transport/flight. However, although there are also numerous publications about military secondary transports of primarily traumatological treatment cases from combat zones (10) or for special diseases, such as COVID-19 in the context of the current pandemic (11, 12), the number of scientific studies on longer secondary transports of non-traumatological intensive care patients by fixed-wing aircraft is rather limited.

Numerous nations have modified widebody aircrafts operated by their Air Forces for transporting patients requiring intensive medical treatment (13–16). In addition of being used to transport soldiers back from operational areas (10), these aircrafts are also employed in humanitarian aid missions in the event of natural disasters or major incidents and for the continued treatment of patients from crisis regions in the context of political agreements.

During intensive care (long-distance) transports, the physical conditions on board the fixed-wing aircraft result in additional aggravating conditions for the patients and the crews. The cruising altitude, the associated gas laws and the forces acting on the aircraft play an important role in this context. As the altitude increases, the atmospheric pressure inside the pressurized cabin also decreases, amounting to only about 80% of that at sea level in a commercial airliner flying at cruising altitude (17). This has implications especially for patients suffering from respiratory diseases (18, 19). All medical devices and aids filled with gases are affected as well (13). Here, the decreasing pressure leads to an increase in volume. This means that, among other things, all drains and drainage tubes must be opened during takeoff and landing (3) so that appropriate pressure equalization can take place. Due to constant climbs and descents, volume decreases or increases can also occur frequently during flight (13). Also, this has an impact on the ventilation situation of the transported patient, and it is general consensus, that patient should be intubated prior to transportation is appropriate if they have any risk of losing airway patency (20). In addition, acceleration forces, vibrations and turbulence, especially during takeoff and landing, affect the patients, making in-flight patient care more difficult for medical staff (3). In summary, this environment is unfavorable for already critically ill intensive care patients during their secondary transport. Scientific findings can help to optimize transport for this patient group.

Aim of this study is an analysis of vital and ventilation changing parameters of intubated patients on long-duration secondary transported fixed-wing aeromedical evacuation (AE) flights.

## 2. Methods

To this end, we evaluated the AE register of the German Air Force Center of Aerospace Medicine as of 30 March 2021. This included the AE flights conducted by the Special Air Mission Wing of the Federal Ministry of Defense with various aircraft types (since 2002) and the AE flights conducted by 62nd Air Transport Wing on the Airbus A-400M “Atlas” (since 2018). The AE register records all patients transported by AE aircrafts of the German Armed Forces (Bundeswehr) for medical reasons, i.e., soldiers flown from various countries of deployment to Germany for further treatment as well as civilians transported within the framework of humanitarian aid missions. To analyse the change of vital and ventilation parameters of intubated patients on long-duration secondary transported fixed-wings AE flights we included for the presented evaluation all patients who were flown in the context of a humanitarian aid mission and were intubated and ventilated at the end of the flight. This required an evaluation of the patient data available in the AE register. These are the data documented by the attending physicians in the respective intensive care transport record sheets from the German Interdisciplinary Association for Intensive Care and Emergency Medicine e.V. (DIVI record) during transport. To centrally record these AE flights for a statistical and scientific evaluation, the DIVI records were compiled in a Microsoft Access database and entered in the statistical software IBM SPSS 24 for Microsoft Windows (SPSS Inc., IL, USA) for later analysis. After a check for plausibility, they were anonymized and subjected to a descriptive statistical evaluation.

All figures are indicated as medians with a range (minimum and maximum) or interquartile ranges. Differences between the beginning of the flight and the end of the flight were analyzed using the Wilcoxon rank sum test for related samples or the chi-squared test with a primary significance level of p < 0.05. For this purpose, we only used the data of patients for whom the respective parameters were available both before and after the flight.

Our analysis was carried out as part of the ministerial research mission of the German Air Force Center of Aerospace Medicine. No additional medical, diagnostic or therapeutic procedures were necessary for this study. It involved only a retrospective evaluation of anonymized medical record data. The data protection officer in charge approved the use of anonymized medical data for the scientific evaluation. In accordance with the requirements of the Ethics Committee of the North Rhine Medical Association and the decision of the Ethics Committee of the Medical Faculty of the Otto von Guericke University of Magdeburg, a formal assessment was not required for the analysis as it is a purely retrospective data analysis.

## 3. Results

**Figure 1:**
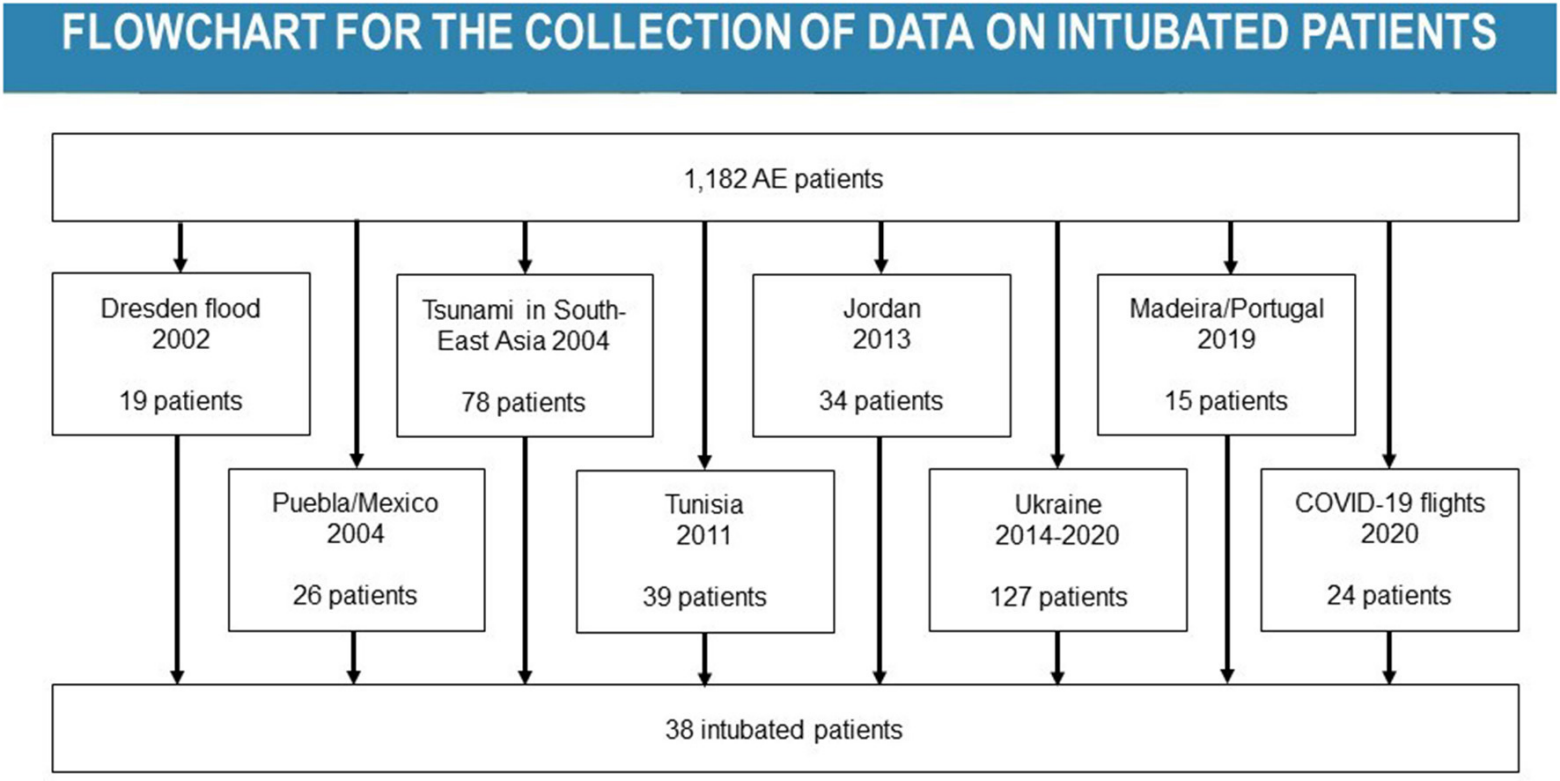
Overview of all cases of intubated and ventilated patients considered for the study. AE, aeromedical evacuation.

In this study, we analyzed 1,182 cases of AE patients recorded in the AE register from 2002 to 2021 (as of 30 March 2021). Of these, 362 patients (31%) had been transported within the context of humanitarian aid missions. Of these 362 patients, 38 had been intubated and ventilated. An overview of the methodological approach is shown in Figure 1.

According to the AE registry data, the 38 intubated and ventilated patient cases were transported on eleven different flights. Ten of these flights were performed with an Airbus A-310 MRTT, and another flight was flown with an Airbus A-400M as part of a humanitarian aid mission. All patients were transported by secondary transport after initial treatment in local hospitals. A large proportion of the patients were flown in the context of COVID-19 humanitarian aid missions in the spring of 2020 (*n* = 24) and during the 2002 flood disaster in eastern Germany when intensive care patients had to be transferred (interhospital transport) (*n* = 11). The three remaining patients were evacuated to Germany for further treatment after events leading to traumas (tsunami disaster in 2004 and bus accident on Madeira in 2019). Table 1 shows a list of all flights on which intubated and ventilated patients were transported in the context of humanitarian aid missions.

**Table 1:** Overview of flights of all intubated and ventilated patients with DIVI records in the period 2002–2021 (in chronological order).

**No**.	**Date of mission**	**Patients**	**Type of aircraft**	**From/to**	**Reason**	**Number of flights**
1–3	15 Aug 2002	11	A-310 MRTT	Dresden–Berlin	Elbe flood	3
4	30 Dec 2004	1	A-310 MRTT	Phuket–Cologne/Bonn	Southeast Asia tsunami victims	1
5	20 Apr 2019	2	A-310 MRTT	Madeira–Cologne/Bonn	Madeira bus accident	1
6	28 Mar 2020	5	A-310 MRTT	Bergamo–Cologne/Bonn	COVID-19 hum. aid	1
7–8	29 Mar 2020	7	A-310 MRTT	Bergamo–Cologne/Bonn	COVID-19 hum. aid	2
9	29 Mar 2020	2	A-400M	Strasbourg–Stuttgart	COVID-19 hum. aid	1
10	01 Apr 2020	4	A-310 MRTT	Bergamo–Cologne/Bonn	COVID-19 hum. aid	1
11	03 Apr 2020	6	A-310 MRTT	Bergamo–Cologne/Bonn	COVID-19 hum. aid	1
		**38**				**11**

Hum. aid, humanitarian aid; MRTT, multi-role transport tanker.

Of the 38 patients, 11 were female (29%) and 27 were male (71%). The median age of the patients was 62.4 years (range: 29–80.8). Of the 38 patients, 32 had documented pre-existing medical diseases, which corresponds to 84%. The most common pre-existing disease was overweight at 34%. A complete list of all pre-existing diseases can be found in Table 2.

**Table 2:** Documented pre-existing medical diseases of the patient group, sorted alphabetically; due to multiple entries, the total number of pre-existing conditions is greater than the number of patients; however, the percentage information refers to the total number of 38 patients.

**Documented pre-existing medical diseases**	***n* (%)**
Accompanying pneumonia	4 (11 %)
- MRSA	3 (8 %)
- Pneumococci	1 (3 %)
Acute renal failure	1 (3 %)
Adrenal insufficiency	1 (3 %)
Aneurysm, cond. after coiling	1 (3 %)
Appendectomy, cond. after	1 (3 %)
Arterial hypertension	7 (18 %)
Bronchial asthma	4 (11 %)
Cholecystectomy, cond. after	1 (3 %)
Chronic hepatitis C	1 (3 %)
Chronic lymphatic leukemia	1 (3 %)
*Chronic obstructive pulmonary disease*	1 (3 %)
Chronic renal insufficiency	1 (3 %)
Chronic sinusitis	1 (3 %)
Coronary heart disease	1 (3 %)
Depression	1 (3 %)
Diabetes mellitus	4 (11 %)
Hypercholesterolemia	2 (5 %)
Hysterectomy, cond. after	1 (3 %)
Intracoronary ascending aortic replacement with valve-carrying conduit in case of aortic dissection, cond. after	1 (3 %)
Left hemicolectomy, cond. after	1 (3 %)
Malignant melanoma, cond. after	1 (3 %)
Myocardial infarction, cond. after	1 (3 %)
Nephrolithiasis	1 (3 %)
Nicotine abuse	2 (5 %)
Obesity (BMI ≥ 30 kg/m^2^)	3 (8 %)
Obstructive sleep apnea syndrome	1 (3 %)
Osteoporosis	1 (3 %)
Overweight (BMI ≥ 25 kg/m^2^)	13 (34 %)
Perpetual arrhythmia	1 (3 %)
Perpetual bradyarrhythmia	1 (3 %)
Pleural effusion, cond. after	1 (3 %)
Renal insufficiency	2 (5 %)
Schizoaffective disorder	1 (3 %)
Sepsis	1 (3 %)
Sigmoid polyp	1 (3 %)
Spinal surgery, cond. after	1 (3 %)
Thyroid nodules	1 (3 %)
Trigeminal neuralgia, b.s.	1 (3 %)
Wertheim's operation, cond. after	1 (3 %)

b.s., on both sides; BMI, body mass index; cond. after, condition after.

When the patients were taken over, the median blood pressure was 115/64 mmHg (range: 70–160 mmHg/30–85 mmHg); at the end of the flight, it was 115/70 mmHg (range: 80–170 mmHg/35–160 mmHg). The median heart rate was 90 beats/min (range: 60–135 beats/min) at takeover and 82 beats/min (range: 45–162 beats/min) after the flight. Of the patients, 16 (42%) had catecholamines administered and 20 (53%) did not require catecholamines; for two patients (5%), no data were available. After the flight, the number of patients requiring catecholamines increased to 23 while the number of patients not requiring catecholamines amounted to eleven; in the case of four patients, the DIVI record did not contain any information on catecholamine administration.

At the beginning of the transport, 24 patients were ventilated with bilevel positive airway pressure (BIPAP), three patients with pressure-controlled ventilation (PCV) and two others with continuous mandatory ventilation (CMV). Two of the patients were intubated during the flight and had spontaneous breathing before the flight. One of these patients had been flown after the Southeast Asia tsunami in 2004 with a complex traumatic injury pattern (femoral fracture and pelvic fracture) and soft tissue infection with sepsis and the second patient had been transported after the Madeira bus accident with traumatic injury also of the lung and with several bone fractures (left humeral shaft fracture, right humeral fracture, right rib fracture). For the remaining seven patients, the type of ventilation used had not been documented. After the flight, BIPAP ventilation was documented in 25 patients, CMV in one patient and PCV in two others. This showed a significant change in ventilation modes with a *p*-value of *p* < 0.001. At the start of the flight, the median oxygen saturation (SaO_2_) was 98% (range: 42–100%) and 25 (66%) of the patients required ventilation with a positive end-expiratory pressure (PEEP) > 8 cmH_2_O. After the flight, 28 patients (74%) required a PEEP > 8 cmH_2_O, which is a statistically significant change (p < 0.001). The median of all PEEP values was 10 cmH_2_O (range: 4–15 cmH_2_O). After the flight, the median for SaO_2_ was 97% (range: 88–100%) and the PEEP was 12 cmH_2_O (range: 4–18 cmH_2_O). The median respiratory rate was 15/min both before and at the end of the flight, with only the range changing from 8–30/min to 10–30/min. The median value for end-expiratory carbon dioxide (etCO_2_) increased from 41 mmHg (range: 6–94 mmHg) to 43 (range: 5–58 mmHg); at the same time, the median respiratory minute volume increased from 8 l/min (range: 5.5–15 l/min) to 9 l/min (range: 4.5–13.2 l/min). Except for the ventilation modes, the fraction of inspired oxygen (FiO_2_) and PEEP > 8 cmH_2_O, none of the values showed changes with a statistically significant difference (p > 0.05). Although the median FiO_2_ remained constant at 0.60 during the entire flight, there was a statistically significant difference (p = 0.029) as the overall FiO_2_ in the entire population decreased (recognizable by a reduction in the 25th and 75th percentiles). The 25th percentile was 0.50 at the beginning of the flight and decreased to 0.40 in the course of the flight, and the 75th percentile decreased from 0.80 to 0.71. A detailed list of the statistical evaluation of all parameters can be found in Table 3.

**Table 3:** Circulatory parameters, ventilation parameters and results of the arterial blood gas analysis of the transported patient group at the beginning and end of the flight.

	***n***	**Before the flight**		**After the flight**		***p***
		**Indicated as number** ***n*** **[%] or median**	**Range**	**Indicated as number** ***n*** **[%] or median**	**Range**	
**Blood pressure**						
Systolic [mmHg]	34	115	(70–160)	115	(80–170)	0.454
Diastolic [mmHg]	33	64	(30–85)	70	(35–160)	0.876
Heart rate [/min]	31	90	(60–135)	82	(45–162)	0.442
**Circulatory condition**						0.138
Stable		21		8		
Unstable		5		1		
Not spec.		12		29		
**Catecholamine req**.						0.117
Yes		16		23		
No		20		11		
Not spec.		2		4		
**Ventilation modes**						**< 0.001**
Spontaneous breathing*		2		-		
BIPAP		24		25		
CMV		2		1		
PCV		3		2		
Not spec.		7		10		
PEEP > 8 cmH_2_O		25		28		
**Ventilation parameters**						**< 0.001**
SaO_2_ [%]	34	98	(42–100)	97	(88–100)	0.556
etCO_2_ [mmHg]	28	41	(6–94)	43	(5–58)	0.092
RR [/min]	32	15	(8–30)	15	(10–30)	0.406
I:E	30	1:1.25	(1:1–1:3)	1:1.4	(1:1–1:3)	0.368
RMV [l/min]	29	8	(5.5–15)	9	(4.5–13.2)	0.668
FiO_2_	34	0.60	(0.3–1)	0.60	(0–1)	**0.016**
PIP [cmH_2_O]	21	27	(18–38)	27	(16–34)	0.489
PEEP [cmH_2_O]	33	10	(4–15)	12	(4–18)	0.216

BIPAP, bilevel positive airway pressure; CMV, continuous mandatory ventilation; not spec., not specified; PCV, pressure-controlled ventilation; PEEP, positive end-expiratory pressure; SaO_2_, oxygen saturation; etCO_2_, end-expiratory carbon dioxide value; RR, respiratory rate; RMV, respiratory minute volume; FiO_2_, fraction of inspired oxygen; PIP, peak inspiratory pressure.

^*^Patients were intubated and ventilated during the flight. Significance values are highlighted in bold.

## 4. Discussion

The present analysis of the secondary transport of a total of 38 intubated and ventilated patients by means of medium-range and long-range intensive care transport aircraft showed that the ventilation status of the patients partly changed during the transport. Two patients had to be intubated during transport (initially they were still breathing spontaneously), and there were significant changes in the necessary positive end-expiratory pressure (PEEP) and the fraction of inspired oxygen (FiO_2_). From our knowledge this is one of the first analysis that have examined this group of patients during long-distance secondary fixed-wing aircraft transport.

The FiO_2_ value decreased in the course of the flight, which generally indicates an improving ventilation status. The reason for this change cannot be explained by the analyzed data, especially an increase of FiO_2_ could be expected because of the lower partial pressure of oxygen on flight level. It is known that also in modern aircraft the arterial oxygen saturation decresed because of the lower cabin pressure inside the aircraft (20, 21). However, especially the secondary transports in the context of humanitarian aid missions repeatedly showed that the local health care systems clearly had exceeded their capacity limits, which meant that the initial therapy was no longer optimal even before the handover (12, 22) and measures had to be taken to optimize patient care before the start of the flight. Thus, all patients undergo a standardized flight preparation what is a standard in AE missions (23). It can be assumed that, in addition, constant monitoring and necessary adjustments of the medical therapy during the AE flights, like recommended in the scientific literature (9), ensured the patients' stable ventilation status. The observed changes in the ventilation parameters are therefore less due to the aerophysiological conditions [air pressure 20% lower than at sea level (24)] than to constant patient monitoring and the fact that the ventilators used can sufficiently compensate for the reduction in oxygen and air pressure (25).

The significant change in the ventilation mode could be explained by the personal preferences of the medical staff instead of challenges in the ventilation parameters of the individual patient. This is already described in the scientific literature (9). In addition, neither the pressure-controlled nor the volume-controlled ventilation impacts the outcome of the patient, so, its use should be guided by preference and experience (26). In addition, it must be taken into account that the AE flights were flown as carefully as possible depending on the mission scenario and the condition of the patients (e.g., adjustment of the cruising altitude to increase cabin pressure, avoiding unnecessary sudden flight maneuvers). However, this approach leads to higher fuel consumption decreases their range, and increases the probability of turbulence, which over all may result in stopovers with increase overall travel time (27). It is therefore always necessary to consider whether a possible improvement of the patients' condition through a possibly more careful transport is advantageous given the longer transport time. Furthermore, the resulting (additional) burden on medical and flight personnel must be taken into account (27).

However, the fact that two of the 38 patients had to be intubated during the flight shows that a deterioration of the ventilation status can occur during AE transports regardless of the pure ventilation parameters. Since intubation and mechanical ventilation of patients are avoided wherever possible, it is not surprising that elective intubation had not been performed on these patients before the AE flights when the ventilation status was still stable. Although in-flight intubation is an exception (only these two out of a total of 1,142 patients in the AE database had to be intubated in flight), it is a particular challenge for the medical crew as the procedure must be carried out under significantly more difficult conditions with the associated increased risks. Because of this patient should be intubated prior to transportation is appropriate if they have any risk of losing airway patency (28).

In contrast to the ventilation parameters, the circulatory parameters did not show any changes. Neither the blood pressure nor the heart rate changed significantly during the flight. This may be due to the optimization of the therapy before the transports and the measures during the AE flights. On the other hand, there was an increase in the number of patients requiring catecholamines. It is no longer possible to determine in the retrospective analysis whether the increasing number of patients requiring catecholamines was necessary because of cardiovascular problems or because of the deepening of the anesthesia due to the flight-related stressors. All patients got a sedation during anesthesia to provide further protection from the aerophysiological stressors occurring during AE flights.

The present study has some strengths and limitations. For example, the data analysis is based on an extensive database comprising almost two decades of AE missions. This made it possible to draw on a very large number of AE patients in order to be able to consider as large a patient group as possible. Particularly with regard to the humanitarian aid missions, which were flown almost exclusively by the aircraft of the Special Air Mission Wing of the Federal Ministry of Defense, it can be said that all patients transported in this period were recorded/considered in the study.

Another strength of the study is that the existing DIVI records contain a large amount of useful information for the analysis of the ventilation status and the circulatory condition of the patients, allowing an accurate analysis of these parameters.

However, the quality of documentation in the available DIVI records is one of the limitations of the present study. In some cases, the forms had not been completely filled out or were difficult to read. Another weakness is that the present data analysis is based only on the available records; other documents (e.g., doctors' letters, transport request documents, etc.) could not be considered because they are no longer available.

By reducing the sample to patients transported in the context of humanitarian aid missions, intubated and ventilated military patients are missing. This selection was chosen to standardize the patient population and to include the widest possible range of pre-existing diseases and patients of different ages and genders. Especially military patients are mostly young, healthy and male due to military requirements. Furthermore, these patients in particular are affected by severe multiple injuries due to combat action, making them a very special patient population.

Despite the comprehensive data analysis, the population of 38 patients in total is nevertheless to be regarded as rather small, which limits the statistical significance. A bigger study cohort should be collected to increase the scientific value of this analysis, it is possible also in a multi-national database analysis.

In summary, it can be stated that during AE flights the ventilation status of intubated patients deteriorated slightly whereas the circulatory parameters did not change during the flights. Acceleration forces, especially during takeoff and landing, (unexpected) turbulence and restrictions due to the cruising altitude make treatment conditions more difficult. Within the scope of this study, all intensive care transport records of the past 20 years were evaluated and analyzed with regard to a possible change in vital signs and ventilation status. Based on the findings gained in this study, existing measures can be improved for future transports of intubated intensive care patients, which will result in optimized patient care.

## Data availability statement

The data that support the findings of this study are available from the Federal Ministry of Defense. Data are available on reasonable request. Requests to access the datasets should be directed to Ministry of Defense, info@bmvg.de.

## Ethics statement

Ethical review and approval was not required for the study on human participants in accordance with the local legislation and institutional requirements. Written informed consent for participation was not required for this study in accordance with the national legislation and the institutional requirements.

## Author contributions

MM and SS were responsible for the study protocol. JP, MM, and VJ for the data collection. JP, MM, and SS did the statistical evaluation and interpretation of the data. JP and MM was the major contributors in writing the manuscript. All authors have read and agreed to the published version of the manuscript.
